# Quantification of Inter-Limb Symmetries With Rate of Force Development and Relaxation Scaling Factor

**DOI:** 10.3389/fphys.2021.679322

**Published:** 2021-06-21

**Authors:** Darjan Smajla, Jure Žitnik, Nejc Šarabon

**Affiliations:** ^1^Faculty of Health Sciences, University of Primorska, Izola, Slovenia; ^2^Human Health Department, InnoRenew CoE, Izola, Slovenia; ^3^Andrej Marušič Institute, University of Primorska, Koper, Slovenia; ^4^S2P, Science to Practice, Ltd., Laboratory for Motor Control and Motor Behavior, Ljubljana, Slovenia

**Keywords:** rapid movement, asymmetry, force, relaxation, basketball, tennis, rapid strength

## Abstract

The inter-limb (a)symmetries have been most often assessed with the tests that quantify the maximal muscle capacity. However, the rapid force production and relaxation during submaximal tasks is equally important for successful sports performance. This can be evaluated with an established rate of force development and relaxation scaling factor (RFD-SF/RFR-SF). The aims of our study were (1) to assess the intra-session reliability of shortened RFD-SF/RFR-SF protocol and its absolute and symmetry outcome measures, (2) to compare the main absolute RFD-SF/RFR-SF outcome measures (slopes of RFD-SF and RFR-SF: k_RTD–SF_ and k_RFR–SF_, theoretical peak RFD/RFR: TP_RFD_ and TP_RFR_) across gender and sports groups, and (3) to compare inter-limb symmetries across gender and sports groups for main outcome measures (k_RFD–SF_, k_RFR–SF_, TP_RFD_, and TP_RFR_). A cross-sectional study was conducted on a group of young health participants (basketball and tennis players, and students): 30 in the reliability study and 248 in the comparison study. Our results showed good to excellent relative and excellent absolute reliability for the selected absolute and symmetry outcome measures (k_RFD–SF_, k_RFR–SF_, TP_RFD_, and TP_RFR_). We found significantly higher absolute values for k_RFD–SF_ and TP_RFD_ in males compared to females for the preferred (k_RFD–SF_: 9.1 ± 0.9 vs. 8.6 ± 0.9/s) and the non-preferred leg (k_RFD–SF_: 9.1 ± 0.9 vs. 8.5 ± 0.8/s), while there was no effect of sport. Significantly lower symmetry values for k_RFR–SF_ (88.4 ± 8.6 vs. 90.4 ± 8.0%) and TP_RFR_ (90.9 ± 6.8 vs. 92.5 ± 6.0%) were found in males compared to females. Moreover, tennis players had significantly higher symmetry values for k_RFR–SF_ (91.1 ± 7.7%) and TP_RFR_ (93.1 ± 6.0%) compared to basketball players (k_RFR–SF_: 88.4 ± 8.7% and TP_RFR_: 90.9 ± 6.7%) and students (k_RFR–SF_: 87.6 ± 8.7% and TP_RFR_: 90.5 ± 6.7%). Our results suggest that the reduced RFD-SF/RFR-SF protocol is a valuable and useful tool for inter-limb (a)symmetry evaluation. Differences in symmetry values in k_RFR–SF_ and TP_RFR_ (relaxation phase) were found between different sports groups. These may be explained by different mechanisms underlying the muscle contraction and relaxation. We suggest that muscle contraction and relaxation should be assessed for in-depth inter-limb symmetry investigation.

## Introduction

Comparing the ability of one limb in contrast to another is a popular research topic from various aspects. Inter-limb asymmetries have been most commonly studied in athletes in relation to sports performance (Sato and Heise, 2012; Lockie et al., 2014; Dos’Santos et al., 2017; Bishop et al., 2018b; Madruga-Parera et al., 2019) and injury risk (Croisier et al., 2008; Lehance et al., 2009; Green et al., 2018). The inter-limb asymmetries have been most often assessed using tests requiring maximal performance such as strength (Fousekis et al., 2010), jumping (Bishop et al., 2020), and change of direction speed tests (Dos’Santos et al., 2017).

To quantify the maximal capacity of the quadriceps muscle, commonly isometric and isokinetic testing protocols are used (Wilson and Murphy, 1996; Croisier et al., 2007; Maffiuletti et al., 2007). Maffiuletti et al. (2010) suggested that rapid force production during voluntary tasks is better related to specific sports performance than maximal force. Furthermore, it is well known that rapid force production is typically required in most sports-specific movements. Capacity of the neuro-muscular system for rapid strength production has been conventionally quantified by rate of force development (RFD) during maximal force production levels. The RFD during maximal voluntary contraction (MVC) is conventionally used to evaluate rapid strength (Knezevic et al., 2014; Mirkov et al., 2017), but, in various sports situations there is a demand for quick submaximal force production, especially in tasks that require precision (Thorlund et al., 2009).

To evaluate inter-limb differences for rapid force production during different submaximal exercises, the rate of force development scaling factor (RFD-SF) was proposed as a measure with higher ecological validity than the RFD (Boccia et al., 2018). In the RFD-SF protocol, the participant needs to perform rapid isometric contractions corresponding to different submaximal percentages of the previously assessed maximal force during MVC. Previous methodological studies recommended performing at least 120 rapid isometric pulses at different force amplitudes for a valid RFD-SF assessment (Bellumori et al., 2011; Djordjevic and Uygur, 2017). The RFD generated during each rapid isometric pulse provides a measure of the rapid force production ability at each of different submaximal amplitudes. The relationship between peak forces and the corresponding RFDs is represented by the slope of these relationships, specifically the slope of the regression line interpolated on the peak force: RFD scatter plot (k_RFD–SF_). This measure enables the investigation of inter-limb asymmetries at movement initiation and quickness of force production, which has been investigated only by two studies to date (Boccia et al., 2018; Smajla et al., 2020). Smajla et al. (2020) showed that leg preference has no effect on k_RFD__–SF_, while Boccia et al. (2018) reported that k_RFD__–SF_ has been shown as a more sensitive tool for identifying inter-limb asymmetry compared to a classical isokinetic test (Boccia et al., 2018). However, both studies were conducted on a small number of participants (up to 40). Recently, one of the studies showed that to assess the ability of quick force relaxation of submaximal muscle forces, the rate of force relaxation scaling factor (RFR-SF) can also be determined as part of the RFD-SF protocol (Mathern et al., 2019). The slope of RFR-SF (k_RFR–SF_) represents the regression line interpolated between peak force and the respective rate of force relaxation. RFR-SF has been shown to be impaired in people with knee osteoarthritis (Šarabon et al., 2020a) and multiple sclerosis (Uygur et al., 2020). It is well acknowledged that adequate muscle relaxation is very important for efficient performance of quick muscle actions (Kato et al., 2019). Moreover, there is clear evidence that strength, power, and speed abilities differ between sports due to specific demands (Šarabon et al., 2020b). However, there are no studies that have compared neuromuscular abilities at a submaximal level between sports and gender or assessed them from the perspective of inter-limb asymmetries. It has been previously shown that k_RFD__–SF_ and k_RFR–SF_ are independent of muscle size (Bellumori et al., 2011; Mathern et al., 2019). Consequently, gender differences are not expected to be found. The main outcome measures of the RFD-SF/RFR-SF protocol can provide additional insight into neuromuscular function as k_RFD–SF_ is mainly dependent on neuromuscular activation mechanisms (Folland et al., 2014), whereas k_RFR–SF_ is mainly influenced by intrinsic properties of the muscle (Mathern et al., 2019). For this reason, such assessment can be valuable for inter-limb asymmetries exploration. Despite a large number of studies that have assessed the magnitude of inter-limb asymmetries using different measures (Sato and Heise, 2012; Maloney et al., 2016; Bishop et al., 2019c; Dos’Santos et al., 2019), there is limited evidence on the variation in inter-limb asymmetries across athletes in different sports.

In our previous work, we found the reduced RFD-SF/RFR-SF protocol (36 pulses) to be reliable for quantifying neuromuscular abilities to quickly produce and relax muscle force during submaximal contraction compared to the standard protocol (100–125 pulses), while introducing a new outcome measure in the form of theoretical maximal RFD/RFR (TP_RFD_ and TP_RFR_) (Smajla et al., 2021b). Therefore, the aims of our study were as follows. First is to assess the intra-session reliability of the absolute RFD-SF/RFR-SF outcome measures and the inter-limb symmetries of the reduced protocol. We hypothesized that all outcome measures would have good to excellent reliability with the exception of the y-intercept. Second is to compare the absolute main outcome measures of interest (k_RFD__–SF_, k_RFR__–SF_, TP_RFD_, and TP_RFR_) across gender and sports groups (basketball players, tennis players, and students from Faculty of Sports). We hypothesized that similar results would be found in males and females, while significant differences between sports groups (basketball players, tennis players, and students of Faculty of Sports) would be found for the main outcome measures. Third is to compare inter-limb symmetries across genders and sports groups for main outcome measures of interest (k_RFD–SF_, k_RFR–SF_, TP_RFD_, and TP_RFR_). We hypothesized that inter-limb symmetries calculated for main outcome measures would not differ between genders, while lower symmetry would be found in basketball players compared to students and tennis players.

## Materials and Methods

### Participants

A total of 248 (152 male and 96 female) participants volunteered for the cross-sectional study. Reliability analyses were conducted on a subsample group, while the differences in absolute and symmetry analyses were conducted on the entire sample (overall sample) (Table 1). Participants from basketball and tennis were included in the study if they had a training history of at least 3 years in their sports and a training frequency of at least two sessions per week in the last year. The students from Faculty of Sports were included in the study in case they had not had specific training history in the past 3 years, but they had been physically active at least two times per week in the last year (most involved in fitness and running). The sample for this study was limited to participants who reported no lower-limb injuries or low-back pain in the past 6 months. Participants with any neurological disorders were also excluded from the analysis. Leg side preference was determined by asking participants: “Which leg do you prefer when performing unilateral jumping movements?” All the participants (or their parent/guardian—if participants were under 18 years of age) were informed of the testing procedures and provided an informed consent prior to study participation. Participants were instructed to avoid intense physical activity for at least 48 h prior to testing. The Slovenian Medical Ethics Committee (approval no. 0120-99/2018/5) approved the experiment, which was conducted according to the guidelines of the Declaration of Helsinki.

**TABLE 1:** Characteristics of the participants in the subsample and overall group.

Sample	Group	Gender	*N*	Age (years)	Body height (cm)	Body mass (kg)	Left preferred (*n*)	Right preferred (*n*)	Training history (years)
Subsample		Male	23	16.5 ± 1.0	188.4 ± 6.8	77.8 ± 10.4	20	3	7.6 ± 2.5
		Female	7	17.3 ± 2.1	175.4 ± 7.5	70.0 ± 15.1	6	1	8.6 ± 3.9
Overall	Basketball	Male	77	16.7 ± 1.1	188.0 ± 8.0	79.0 ± 10.7	65	12	7.3 ± 2.2
		Female	40	16.9 ± 1.6	175.3 ± 5.8	70.8 ± 9.6	37	3	6.8 ± 2.5
	Students	Male	25	19.7 ± 0.4	182.5 ± 5.7	75.7 ± 8.2	9	16	8.3 ± 3.8
		Female	25	19.7 ± 0.7	166.9 ± 6.0	59.9 ± 7.8	11	14	8.6 ± 4.4
	Tennis	Male	50	16.1 ± 3.0	177.1 ± 8.4	67.1 ± 10.1	36	14	8.8 ± 3.8
		Female	31	16.3 ± 2.7	169.2 ± 5.9	61.2 ± 8.0	21	10	8.1 ± 3.7

### Study Design, Tasks, and Procedures

**FIGURE 1:**
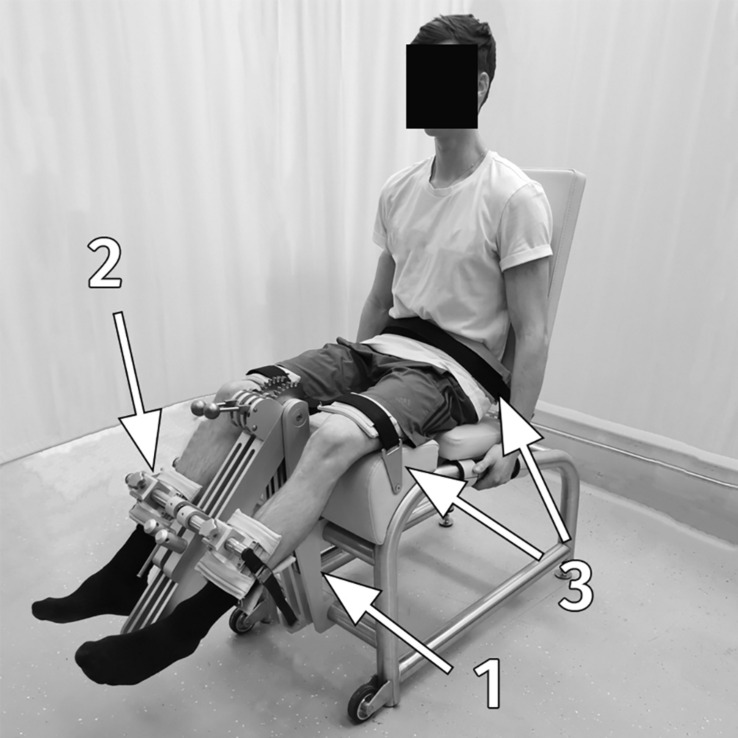
Measurement setup; the subject in the isometric knee dynamometer: **1**, a strain gage force sensor; **2**, shank support; **3**, rigid straps for the pelvis and knee fixation.

Each of the participants performed a knee-extension MVC with the preferred and non-preferred leg in a randomized order prior to performing the RFD-SF/RFR-SF protocol. Although torque was measured, the term force is used in this article to maintain consistency with previous literature (Bellumori et al., 2011; Mathern et al., 2019). For the MVC and RFD-SF/RFR-SF protocols, participants sat in the chair of an isometric knee dynamometer (S2P, Science to Practice, Ltd., Ljubljana, Slovenia; Figure 1; Sarabon et al., 2013). The knee angle was set at 60° of knee flexion (full knee extension equals 0°), while the hip angle was set at 100°. The knee axis was in line with the axis of the lever arm of the dynamometer. The shank support was manually adjusted for each participant so that it was positioned approximately 2 cm above the lateral malleolus. Hip and knee fixation was provided by rigid straps over the pelvis and the knee. For familiarization and warm-up, each participant first performed two graded submaximal contractions at 50, 75, and 90% of the self-estimated maximal voluntary effort. After 3 min of rest, participants performed three maximal voluntary knee extensions with a 30-s rest interval. They were instructed to gradually increase their torque and maintain maximal force for 3–5 s, from which peak knee extension force was determined. Participants then performed 15–20 submaximal rapid contractions and relaxations performed at varying submaximal levels or until they could match the force and perform voluntary force pulses and relaxations with the required intensity. They were instructed to produce the knee extension movement as quickly as possible and then immediately relax. After familiarization, participants in the subsample group performed approximately 25–30 rapid isometric contractions at four different submaximal levels (20, 40, 60, and 80% of previously determined maximal voluntary force), for a total of 100–120 contractions. All other participants performed a reduced RFD-SF/RFR-SF protocol (Smajla et al., 2021b). We aimed for 60 pulses to be performed as some contractions might not be performed properly (slow contraction, pre-contraction of agonist or antagonist inappropriate relaxation) (Grabiner, 1994; Kamimura et al., 2009) and would be excluded from the final analysis. The target force level was displayed on a computer screen in front of the participant performing the protocol as a horizontal line on a graph. Visual feedback on the force generated by the participant during the pulse was also displayed on the screen, while participants were instructed to apply a level of force that matched the horizontal target force level displayed during each pulse. As previously suggested by Gordon and Ghez (1987), participants were instructed to focus on rapid performance rather than attempting to match force levels. There was a 60-s rest interval between two consecutive submaximal levels. Rapid pulses were elicited by an experienced examinator with a verbal command at approximately 4–5-s intervals. The reliability and validity of the RFD-SF/RFR-SF outcome measures were first examined on a subsample of participants (Table 1). To assess the intra-session reliability in the subsample study, two sets of 36 pulses were sampled separately (nine randomly selected repetitions for each submaximal intensity) and used to calculate the outcome measures. Subsequently, all participants performed the reduced RFD-SF/RFR-SF protocol with 36 pulses to obtain the final outcome-measures data.

### Data Processing and Outcome Measures

The signals from the knee dynamometer force transducer (Bending beam load cell 1-Z6FC3, Darmstadt, Germany) were sampled at 1,000 Hz by a custom-made LabView 2015 routine (National Instruments Corp., Austin, TX, United States). A low-pass filter (Butterworth second-order) with a 5-Hz cut-off frequency (Djordjevic and Uygur, 2017) was used for raw data signal filtration. Another custom-made LabView 2015 routine (National Instruments Corp., Austin, TX, United States) routine was used for data analysis. The program routine automatically placed two cursors on the time derivate curve corresponding to peak RFD and peak RFR (1-ms sampling window) of each of the acquired force pulses. One additional cursor was placed on the force curve and depicted the peak force. We performed manual inspection for all signals to verify correct cursor placement. For each pulse, we recorded the magnitudes of peak force, RFD, and RFR (Mathern et al., 2019). The regression parameters were obtained from the relationship between the peak torque and corresponding peak RFD and peak RFR and used as a dependent variable. This relationship was used for the calculation of k_RFD–SF_ (/s) and k_RFR–SF_ (/s) of the regression line (main dependent outcome measures of interest). Other linear regression parameters, r^2^ (r^2^_RFD__–SF_, r^2^_RFR__–SF_), y-intercept [y-int_RFD_ (%MVC/s), y-int_RFR_ (%MVC/s)], and theoretical peak RFD/RFR [TP_RFD_ (%MVC/s), TP_RFR__–SF_ (%MVC/s)] were calculated for each participant. TP_RFD_ and TP_RFR_ represent the newly introduced outcome measures determined by linear interpolation for each participant’s regression line (*y* = *k* × *x* + *n*) solved for *x* = 100 (maximal theoretical peak RFD/RFR). Inter-limb symmetries were calculated for main outcome measures (k_RFD–SF_, k_RFR–SF_, TP_RFD_, and TP_RFR_) in accordance with the previously published equation (Smajla et al., 2021a) (Eq. 1).

$$\mathrm{Symmetry}\left(\%\right)=100-\left(\frac{\mathrm{higher}\,\mathrm{value}-\mathrm{lower}\,\mathrm{value}}{\mathrm{higher}\,\mathrm{value}}\right)\times 100$$

### Statistical Analysis

R Statistical Software (version 4.0.3, R Core Team, R Foundation for Statistical Computing, Vienna, Austria) was used for all the performed statistical analyses. Descriptive statistics of the dependent variables are presented as mean ± standard deviation. In the subsample analysis, the following reliability measures were used to describe intra-session reliability for absolute and symmetry outcome measures: (a) intraclass correlation coefficient (ICC_2,1_), (b) coefficient of variation (CV), and c) typical error (TE) (Hopkins, 2000). ICC_2,1_ estimates and their 95% confidence intervals (CI) were interpreted according to Koo and Li (2015), while CV (within-subject standard deviation method) values of <10% were considered acceptable (Cormack et al., 2008). Prior to analysis, the data distribution for each of the outcome measures in the groups under study was assessed by performing a Shapiro–Wilk test and looking at Q–Q plots of the residuals. The homogeneity of variance was determined with the use of Levene’s test. A two-way analysis of variance (ANOVA) was used to examine the effect of gender and sports on main outcome measures k_RFD–SF_, k_RFR–SF_, TP_RFD_, and TP_RFR_, followed by Bonferroni-adjusted significance tests for *post hoc* pairwise comparisons. The inter-limb symmetry outcome measures were not normally distributed; therefore, a robust two-way ANOVA on trimmed means using the *t2way* function from the WRS2 package (Mair and Wilcox, 2020) was performed for between-group comparisons. When the main effect was found to be significant, the Yuen’s modified test for independent trimmed means (20% level) (Yuen, 1974) with a bootstrap (*n* = 599) was used for *post hoc* group comparisons (*yuenbt* function from the WRS2 package). The effect size for the two-way ANOVA of the main outcome measures was described using partial eta squared ($\eta_{p}^{2}$) with the following interpretation: small (0.01–0.06), medium (0.06–0.14), and large (>0.14) (Cohen, 1992). Classical Cohen’s *d* effect size was calculated for *post hoc* comparisons (Cohen, 1988) of main outcome measures. For determining the magnitude of inter-limb symmetries by *post hoc* comparisons, a robust alternative to the classical Cohen’s *d* effect size with percentile bootstrap 95% CI was calculated (δ_R_) according to Algina et al. (2005). Both Cohen’s *d* and δ_R_ were interpreted as follows: negligible (<0.2), small (0.2–0.5), moderate (0.5–0.8), and large (>0.8). For all statistical tests performed, significance level was set at *p* < 0.05 (two-tailed).

## Results

### Reliability

**FIGURE 2:**
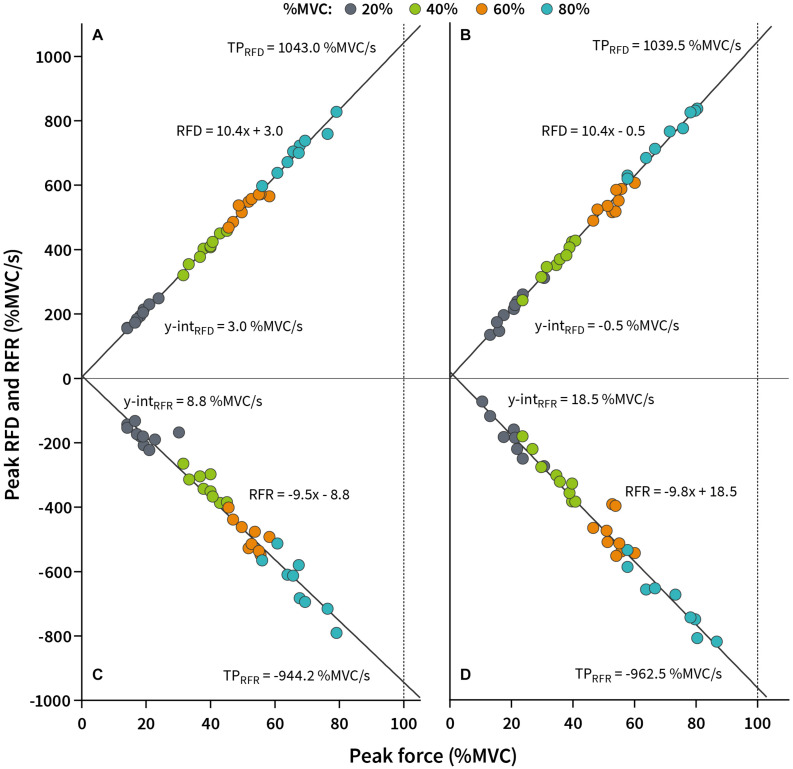
The slope of the rate of force development/relaxation scaling factor (k_RFD–SF_/k_RFR–SF_) of a representative participant interpolated to a data scatter. **(A)** Scatter plot and regression line of the nine randomly selected pulses at each submaximal level used for calculation of the k_RFD–SF_. **(B)** Scatter plot and regression line of the remaining nine randomly selected pulses (after the first selection) at each submaximal level used to calculate the k_RFD–SF_. **(C)** Scatter plot and regression line of the nine randomly selected pulses at each submaximal level used to calculate the k_RFR–SF_. **(D)** Scatter plot and regression line of the remaining nine randomly selected pulses at each submaximal level used for calculation of the k_RFR–SF_. k_RFD–SF_, slope of the rate of force development scaling factor; k_RFR–SF_, slope of the rate of force relaxation scaling factor; y-int_RFD_, y-intercept of RFD-SF; y-int_RFR_, y-intercept of RFR-SF; TP_RFD_, theoretical peak rate of force development; TP_RFR_, theoretical peak rate of force relaxation.

The first set of analysis examined the reliability of absolute outcome measures and outcome measures for inter-limb symmetry. Figure 2 displays the raw pulse data obtained during the RFD-SF/RFR-SF protocol for a single participant in the subsample study and demonstrates differences in outcome measures between randomly selected repetition clusters.

All the subsample reliability statistics are presented in Table 2. Overall, we found good to excellent relative (ICC_2,1_) and excellent absolute reliability (CV) for the selected absolute main outcome measures (k_RFD–SF_, k_RFR–SF_, TP_RFD_, and TP_RFR_) in both the preferred and non-preferred leg. Moderate to excellent relative and excellent absolute reliability was observed for symmetry main outcome measures.

**TABLE 2:** Reliability results for absolute and inter-limb symmetry main outcome measures.

	Outcome measure	Selection 1	Selection 2	ICC (95% CI)	CV	TE
Preferred leg	k_RFD–SF_ (/s)	9.1 ± 1.1	9.1 ± 1.1	0.96 (0.92, 0.98)	2.5%	0.20
	k_RFR–SF_ (/s)	−7.9 ± 1.3	−7.9 ± 1.4	0.94 (0.89, 0.97)	4.2%	0.30
	r^2^_RFD–SF_	0.97 ± 0.02	0.97 ± 0.02	0.67 (0.46, 0.81)	1.3%	0.01
	r^2^_RFR–SF_	0.90 ± 0.09	0.89 ± 0.07	0.82 (0.69, 0.90)	3.6%	0.03
	y-int_RFD_ (%MVC/s)	27.2 ± 37.3	25.8 ± 33.9	0.94 (0.90, 0.97)	*	8.50
	y-int_RFR_ (%MVC/s)	−28.4 ± 43.1	−28.4 ± 45.0	0.91 (0.84, 0.95)	*	13.60
	TP_RFD_ (%MVC/s)	932.8 ± 83.6	934.7 ± 82.9	0.97 (0.94, 0.98)	1.6%	15.20
	TP_RFR_ (%MVC/s)	−815.7 ± 115.2	−816.6 ± 117.9	0.96 (0.94, 0.98)	2.7%	22.40
Non-preferred leg	k_RFD–SF_ (/s)	8.8 ± 1.2	8.9 ± 1.1	0.95 (0.90, 0.97)	2.9%	0.30
	k_RFR–SF_ (/s)	−7.8 ± 1.5	−7.9 ± 1.4	0.91 (0.83, 0.95)	5.7%	0.50
	r^2^_RFD–SF_	0.97 ± 0.02	0.97 ± 0.04	0.82 (0.68, 0.90)	1.5%	0.01
	r^2^_RFR–SF_	0.89 ± 0.11	0.90 ± 0.09	0.83 (0.71, 0.90)	5.0%	0.04
	y-int_RFD_ (%MVC/s)	25.4 ± 30.5	23.3 ± 28.2	0.90 (0.82, 0.95)	*	9.30
	y-int_RFR_ (%MVC/s)	−17.3 ± 50.6	−16.1 ± 50.5	0.87 (0.77, 0.93)	*	18.70
	TP_RFD_ (%MVC/s)	905.3 ± 91.8	912.6 ± 87.5	0.96 (0.93, 0.98)	1.9%	17.50
	TP_RFR_ (%MVC/s)	−793.4 ± 121.7	−804.9 ± 109.1	0.94 (0.89, 0.97)	3.5%	27.90
Symmetry (%)	k_RFD–SF_ (%)	91.6 ± 5.1	92.7 ± 4.6	0.78 (0.63, 0.88)	2.4%	2.20
	k_RFR–SF_ (%)	87.3 ± 10.1	87.6 ± 10.3	0.75 (0.58, 0.86)	5.9%	5.20
	TP_RFD_ (%)	93.9 ± 3.8	94.6 ± 3.6	0.85 (0.74, 0.92)	1.5%	1.40
	TP_RFR_ (%)	90.0 ± 8.0	90.3 ± 8.0	0.79 (0.65, 0.88)	4.1%	3.70

Selection 1, mean (±SD) values for nine randomly selected pulses; Selection 2, mean (±SD) values for nine remaining pulses after the first selection; ICC_2,1_ (95% CI), intraclass correlation coefficient (95% confidence interval); CV, coefficient of variation; TE, typical error; k_*RFD–SF*_, slope of the rate of force development scaling factor; k_*RFR–SF*_, slope of the rate of force relaxation factor; y-int_*RFD*_, y-intercept of RFD; y-int_*RFR*_, y-intercept of RFR-SF; r^2^_*RFD–SF*_, linearity of regression line for RFD-SF; r^2^_*RFR–SF*_, linearity of regression line for RFR-SF; TP_*RFD*_, theoretical peak rate of force development; TP_*RFR*_, theoretical peak rate of force relaxation. * CV not reported as the y-intercept outcome measures contain both positive and negative values.

### Gender and Sports Differences for Absolute Main Outcome Measures

The effect of gender and sports on each of the absolute main outcome measures was examined by conducting a two-way ANOVA test. For both the preferred and non-preferred leg, there was no significant interaction between gender and sports on any of the main outcome measures (Table 3).

**TABLE 3:** Mean (±SD) values of absolute main outcome measures and two-way ANOVA results.

	Outcome measure	Basketball		Students		Tennis		*p*-Value			$\eta_{p}^{2}$		
		**M**	**F**	**M**	**F**	**M**	**F**	**Gender**	**Sport**	**Interaction**	**Gender**	**Sport**	**Interaction**
Preferred leg	k_RFD–SF_ (/s)	9.23 ± 0.93	8.72 ± 0.97	8.88 ± 0.87	8.58 ± 0.97	9.07 ± 0.91	8.49 ± 0.63	**<0.001**	0.178	0.687	0.066	0.014	0.003
	k_RFR–SF_ (/s)	−8.20 ± 1.20	−7.83 ± 1.43	−8.17 ± 0.94	−8.41 ± 1.09	−7.93 ± 1.20	−8.25 ± 0.92	0.959	0.491	0.104	0.000	0.006	0.019
	TP_RFD_ (%MVC/s)	946.5 ± 69.4	900.1 ± 73.7	921.7 ± 63.4	890.6 ± 76.1	936.7 ± 68.1	892.0 ± 50.4	**<0.001**	0.279	0.795	0.086	0.010	0.002
	TP_RFR_ (%MVC/s)	−836.1 ± 98.3	−791.3 ± 132.8	−831.2 ± 70.6	−842.7 ± 93.1	−820.5 ± 102.0	−839.8 ± 78.5	0.386	0.566	0.072	0.003	0.005	0.022
Non-preferred leg	k_RFD–SF_ (/s)	9.03 ± 0.92	8.37 ± 0.89	9.07 ± 0.88	8.47 ± 0.90	9.10 ± 0.80	8.55 ± 0.68	**<0.001**	0.685	0.908	0.108	0.003	0.001
	k_RFR–SF_ (/s)	−7.92 ± 1.39	−7.56 ± 1.04	−8.12 ± 1.16	−8.04 ± 0.93	−7.87 ± 1.16	−8.10 ± 0.89	0.479	0.289	0.242	0.002	0.010	0.012
	TP_RFD_ (%MVC/s)	924.3 ± 71.7	869.1 ± 68.9	934.7 ± 67.9	885.7 ± 70.3	938.1 ± 58.9	896.8 ± 51.7	**<0.001**	0.131	0.780	0.118	0.017	0.002
	TP_RFR_ (%MVC/s)	−807.3 ± 112.6	−761.4 ± 103.0	−827.3 ± 97.7	−815.9 ± 77.1	−813.4 ± 99.1	−832.7 ± 74.7	0.197	0.053	0.093	0.003	0.005	0.022

*M, males; F, females; $\eta_{p}^{2}$, partial eta squared; k_*RFD–SF*_, slope of rate of force development scaling factor; k_*RFR–SF*_, slope of rate of force relaxation factor; TP_*RFD*_, theoretical peak rate of force development; TP_*RFR*_, theoretical peak rate of force relaxation. Bold values indicate significance.*

**FIGURE 3:**
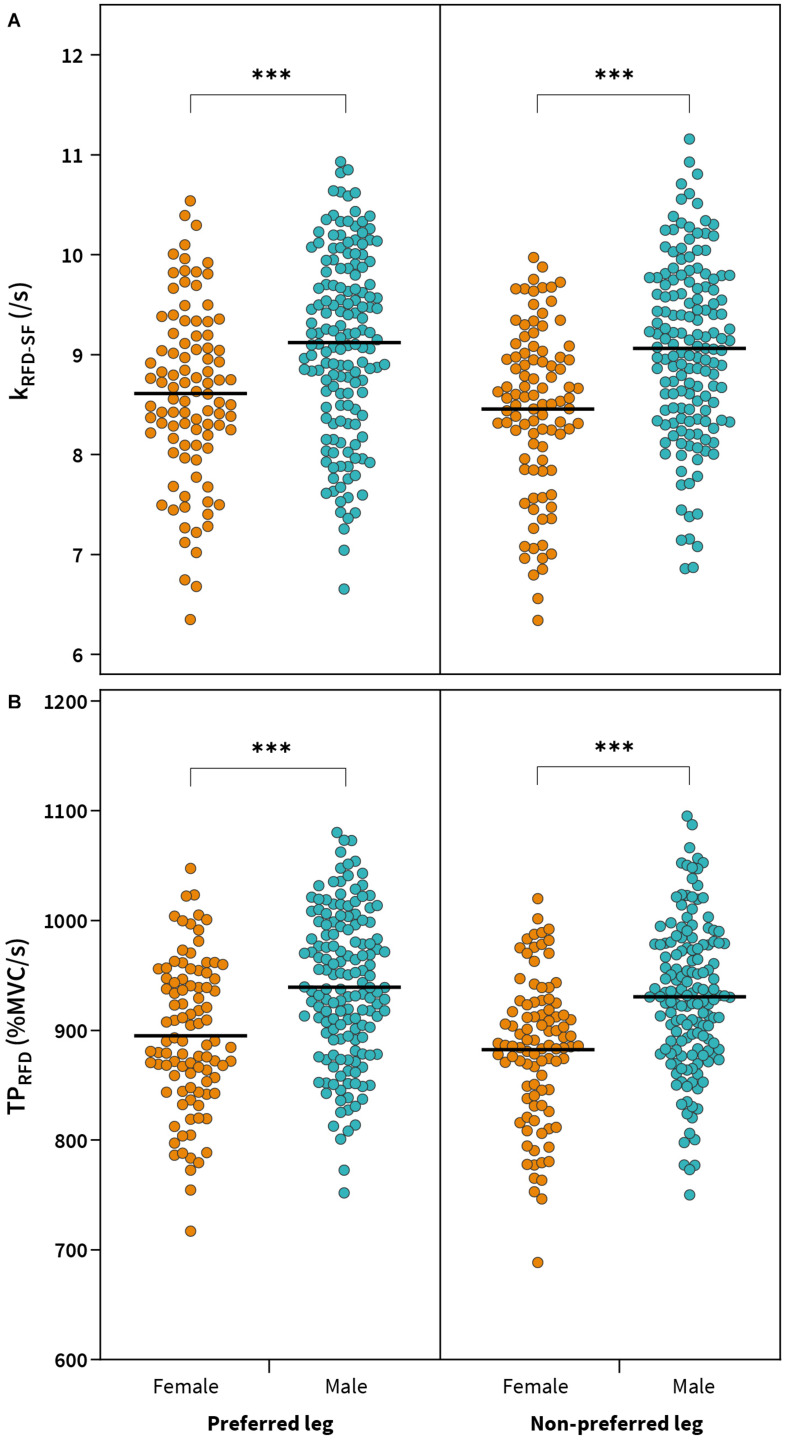
Comparison between females and males for RFD-SF slope (k_RTD–SF_) **(A)** and theoretical peak rate of force development (TP_RFD_) **(B)**. ^∗∗∗^*p* < 0.001.

For the preferred leg, there was a significant main effect of gender on k_RFD–SF_ [*F*_(1,242)_ = 17.03, *p* < 0.001, $\eta_{p}^{2}=0.06$) and TP_RFD_ [*F*_(1,242)_ = 22.69, *p* < 0.001, $\eta_{p}^{2}=0.09$] with higher k_RFD–SF_ (9.1 ± 0.9 vs. 8.6 ± 0.9/s, *d* = 0.57) and TP_RFD_ (939.2 ± 68.2 vs. 895.0 ± 67.2 %MVC/s, *d* = 0.65) found in males compared to females (Figure 3). No main effect of sports on other main outcome measures was found for the preferred leg (Table 3).

For the non-preferred leg, a significant main effect of gender was found for k_RFD–SF_ [*F*_(1,242)_ = 29.33, *p* < 0.001, $\eta_{p}^{2}=0.11$] and TP_RFD_ [*F*_(1,242)_ = 32.29, *p* < 0.001, $\eta_{p}^{2}=0.12$] (Table 3). Males showed significantly higher k_RFD–SF_ (9.1 ± 0.9 vs. 8.5 ± 0.8/s, *d* = 0.71) and TP_RFD_ (931.0 ± 67.0 vs. 882.4 ± 64.7%MVC/s, *d* = 0.73) compared to females (Figure 3), while no significant main effect of sports was observed on other main outcome measures.

### Gender and Sports Differences for Inter-Limb Symmetry Measures

**FIGURE 4:**
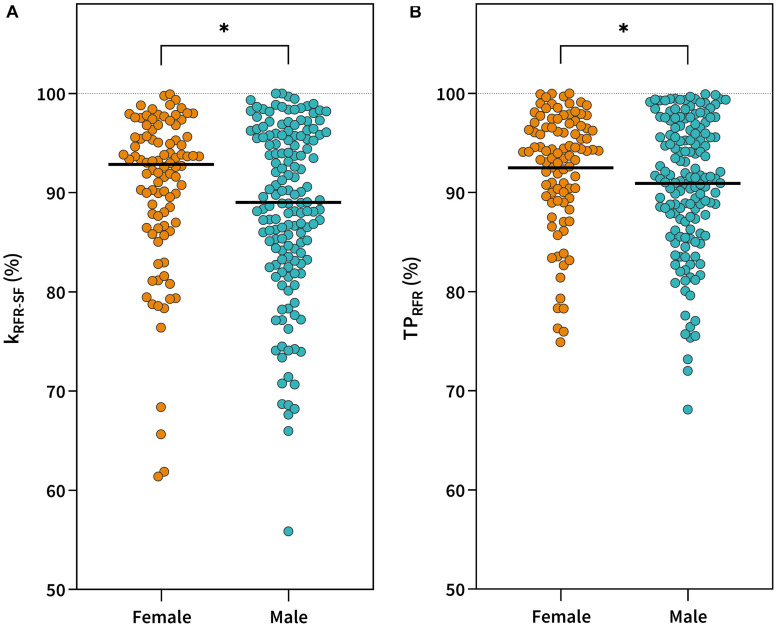
Gender comparison in symmetry values for RFR-SF slope (k_RFR–SF_) **(A)** and theoretical peak rate of force relaxation (TP_RFR_) **(B)**. ^∗^*p* < 0.05.

The effect of gender and sports on each of the main outcome measures was examined by conducting a robust nonparametric two-way ANOVA test. No significant interaction was found between gender and sports for any of the inter-limb symmetry outcome measures (Table 4). A significant main effect of gender was found for inter-limb symmetries in k_RFR–SF_ (*p* < 0.05) and TP_RFR_ (*p* < 0.05). Yuen *post hoc* test revealed lower symmetry values in males for k_RFR–SF_ [88.4 ± 8.6 vs. 90.4 ± 8.0%, *p* ≤ 0.05, δ_R_ = 0.31 (95% CI 0.06–0.54)] and TP_RFR_ [90.9 ± 6.8% vs. 92.5 ± 6.0%, *p* ≤ 0.05, δ_R_ = 0.29 (95% CI 0.04–0.52)] (Figure 4).

**TABLE 4:** Mean (±SD) values of inter-limb symmetry main outcome measures and two-way ANOVA results.

Outcome measure	Basketball			Students			Tennis			*p*-Value		
	M	F	Pairwise	M	F	Pairwise	M	F	Pairwise	Gender	Sport	Interaction
k_RFD–SF_ (/s)	93.23 ± 5.6	92.35 ± 5.4	ns	92.71 ± 5.1	91.41 ± 5.9	ns	93.24 ± 5.2	94.80 ± 5.1	ns	0.814	0.085	0.156
k_RFR–SF_ (/s)	87.74 ± 8.7	89.67 ± 8.6	a*	86.76 ± 8.1	88.57 ± 9.4	b*	90.09 ± 8.8	92.76 ± 5.3	a*, b*	**0.040**	**0.016**	0.848
TP_RFD_ (%MVC/s)	94.81 ± 4.0	94.63 ± 3.6	ns	94.88 ± 3.9	94.02 ± 4.2	ns	95.13 ± 3.8	95.95 ± 3.5	ns	0.742	0.175	0.445
TP_RFR_ (%MVC/s)	90.60 ± 6.9	91.45 ± 6.3	a**	89.55 ± 6.5	91.43 ± 6.8	b*	92.07 ± 6.8	94.67 ± 4.1	a**, b*	**0.033**	**0.010**	0.871

Values are presented as mean ± SD. M, males; F, females; k_*RFD–SF*_, slope of rate of force development scaling factor; k_*RFR–SF*_, slope of rate of force relaxation factor; TP_*RFD*_, theoretical peak rate of force development; TP_*RFR*_, theoretical peak rate of force relaxation; a, Basketball vs. Tennis; b, Students vs. Tennis; *p < 0.05; **p < 0.01; ns, not significant. Bold values indicate significance.

**FIGURE 5:**
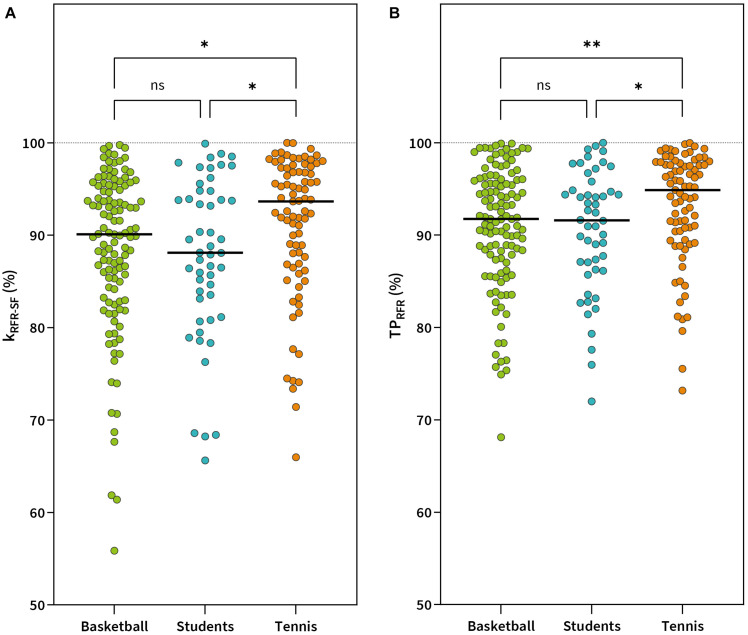
Between sports comparison of symmetry values for RFR-SF slope (k_RFR–SF_) **(A)** and theoretical peak rate of force relaxation (TP_RFR_) **(B)**. ^∗∗^*p* < 0.01; *p < 0.05; ns, not significant.

A significant main effect of sports was also found for k_RFR–SF_ (*p* < 0.05) and TP_RFR_ (*p* < 0.01). Pairwise *post hoc* tests between individual sports groups showed that tennis players had significantly higher inter-limb symmetry in k_RFR–SF_ (91.1 ± 7.7%) compared to basketball players [88.4 ± 8.7, *p* < 0.05, δ_R_ = 0.37 (95% CI 0.12–0.72)] and students [87.6 ± 8.7, *p* < 0.05, δ_R_ = 0.52 (95% CI 0.20–0.97)] while the difference in k_RFR–SF_ symmetries between basketball players and students was not significant (*p* = 0.46) (Figure 5). Significantly higher values for inter-limb symmetry were also found in tennis players for TP_RFR_ (93.1 ± 6.0%) compared to basketball players [90.9 ± 6.7, *p* < 0.01, δ_R_ = 0.41 (95% CI 0.12–0.70)] and students [90.5 ± 6.7, *p* < 0.05, δ_R_ = 0.50 (95% CI 0.12–0.93)] while no significant differences were found between basketball players and students (*p* = 0.69) (Figure 5).

## Discussion

The aim of the present study was to assess the reliability of the reduced RFD-SF/RFR-SF protocol for absolute outcome measures and the inter-limb symmetry measures calculated from the main absolute outcome measures (k_RFD–SF_, k_RFR–SF_, TP_RFD_, and TP_RFR_). Additionally, we investigated differences for the main absolute and symmetry outcome measures across gender and sports. The analysis yielded the following results: (1) good to excellent reliability for the main absolute outcome measures and moderate to excellent reliability for inter-limb symmetry outcome measures; (2) significantly higher absolute values for k_RFD–SF_ and TP_RFD_ in males (both legs) compared to females, while there was no effect of sport; and (3) significantly lower symmetry values for k_RFR–SF_ and TP_RFR_ in males compared to females and significantly higher symmetry values for k_RFR–SF_ and TP_RFR_ for tennis players compared to basketball players and students.

Using two different subsets of pulses (36 pulses), excellent intra-session reliability was calculated for the main absolute outcome measures k_RFD–SF_, TP_RFD_, and TP_RFR_, while good to excellent reliability was found for k_RFR–SF_. As evidenced by a large TE found for outcome measures y-int_RFD_ and y-int_RFR_, the results of this study confirm observations of other authors about the y-intercept negligibility (Casartelli et al., 2014; Bellumori et al., 2017; Djordjevic and Uygur, 2017; Smajla et al., 2021b). Moderate to good reliability (moderate to excellent for TP_RFD_) was observed for the RFD-SF/RFR-SF protocol-based inter-limb symmetry main outcome measures, enabling further comparisons between genders and different sports. The importance of the reliability of the inter-limb symmetry calculation method is highlighted by several previous studies, which observed significant variation in inter-limb symmetry and asymmetry values (Bishop et al., 2018a, 2019b) as poor reliability limits the practical application of study results. Based on the observed results, we can partially confirm our first hypothesis because most of the outcome measures including y-intercepts showed good to excellent ICC_2,1_ values. Not all outcome measures showed satisfactory reliability as poor to good reliability with notably wider 95% CI limits was found for r^2^_RFD–SF_ in the preferred leg and moderate to excellent reliability was found for r^2^_RFR–SF_ (preferred leg), r^2^_RFD–SF_ (non-preferred leg), and r^2^_RFR–SF_ (non-preferred leg). Despite the satisfactory relative agreement (ICC_2,1_) for the y-intercept, high TEs would suggest that the use of this outcome measure is questionable.

Previous studies have suggested that RFD-SF/RFR-SF measures are independent of gender, and muscle size and strength (Bellumori et al., 2011; Djordjevic and Uygur, 2017). On this basis, we hypothesized that there would be no difference in the main absolute outcome measures between males and females. However, the analysis showed a significant effect of gender, as males showed significant greater values for k_RFD–SF_ (9.1 ± 0.9 vs. 8.6 ± 0.9/s) and TP_RFD_ (939.2 ± 68.2 vs. 895.0 ± 67.2%MVC/s) in the preferred and-non preferred leg (k_RFD–SF_: 9.1 ± 0.9 vs. 8.5 ± 0.8/s; TP_RFD_: 931.0 ± 67.0 vs. 882.4 ± 64.7%MVC/s). Prior studies using RFD-SF measures have confirmed a reduction in neuromuscular quickness with aging (Bellumori et al., 2013) and other impairments such as multiple sclerosis (Uygur et al., 2020) and knee osteoarthritis (Šarabon et al., 2020a), while there has been no evidence of the influence of gender on RFD-SF/RFR-SF outcome measures. Males and females in our study did not differ in age or training history which could have an influence on the observed results. When performing RFD-SF/RFR-SF calculations, the RFD is normalized to the participant’s MVC, making this measure independent of muscle size and strength. For this reason, other mechanisms might be responsible for the observed gender differences. In the early phase of force generation, a series of elastic components in the muscle–tendon complex must be stretched, which is related to the electromechanical delay (time from the onset of muscle activation until the onset of joint torque production) (Komi, 1984). Some previous studies have shown that males have a shorter electro-mechanical delay of the knee extensors compared to females (Bell and Jacobs, 1986; Zhou et al., 1995), while the stiffness and hysteresis of the tendon structure are lower in females compared to males (Kubo et al., 2003). This greater extensibility of tendon structures may cause a smaller transmission of contractile force on the joint and consequently on the maximal RFD at different submaximal intensities. This may partially explain greater k_RFD–SF_ and TP_RFD_ in males compared to females. The differences between males and females occurred only for outcome measures that are related to force production (k_RFD–SF_ and TP_RFD_). These measures are mainly dependent on neuromuscular activation mechanisms (Folland et al., 2014) such as double discharges and high firing rates (Klass et al., 2008), in addition to the mechanical properties of the muscle (contractile and connective tissue). Females have been shown to require greater neural drive to achieve fused tetanus during submaximal contractions compared to males (Inglis and Gabriel, 2020), which could further explain the gender differences observed in our study. On the other hand, the force relaxation mainly depends on the intrinsic properties of the muscle (Mathern et al., 2019). Even though it has been previously shown that females have a lower maximal rate of muscle relaxation (Amsterdam et al., 2008), these differences did not affect the RFR-SF outcome measures in our case.

Although we expected to find differences in our main outcome measures, there was no significant effect of sport. Basketball players showed the highest mean values of main outcome measures k_RFD–SF_, k_RFR–SF_, TP_RFD_, and TP_RFR_, while the smallest values were observed in students. However, these differences were not found to be significant. These results and the differences between genders allow us to reject our second hypothesis. This is the first study to evaluate the differences in RFD-SF/RFR-SF outcome measures between different sports. The results indicate that the type of training regimes that are typical of each of our studied groups does not affect the RFD-SF/RFR-SF outcome measures. The differences were expected as one of the previous studies showed that knee extensor strength was greater in tennis players compared to basketball players (Šarabon et al., 2020b). However, this only refers to maximal capacity, whereas in the RFD-SF/RFR-SF protocol force production is evaluated at submaximal force production levels. Another possible explanation could be due to the young age of our sample in which distinct adaptations induced by specific training regimes are not yet apparent.

Inter-limb (a)symmetry quantification has been widely used in sports as it has been associated with sports performance (Bishop et al., 2019a; Madruga-Parera et al., 2019) and injury risk (Murphy et al., 2003; Lehance et al., 2009). Despite a large number of studies on inter-limb (a)symmetries, little is known to date about their variation between different sports. Some prior studies have mainly assessed maximal capacities (e.g., maximal strength, power, and speed) (Dos’Santos et al., 2018; Bishop et al., 2019a; Šarabon et al., 2020b). With the RFD-SF/RFR-SF protocol, we aim to assess rapid force production and relaxation across different ranges of submaximal intensities that are highly relevant to sports performance. To date, there are only two studies that have investigated inter-limb (a)symmetries with RFD-SF protocol, while the evaluation of (a)symmetries during the relaxation phase has not yet been reported. Boccia et al. (2018) showed that the prevalence of asymmetry was greater for k_RFD–SF_ compared to the classical isokinetic test in young soccer players. They found lower symmetry values for knee extensors (82.6 ± 9.1%) compared to male basketball players (93.23 ± 5.6%), male tennis players (92.71 ± 5.1%), and male students (93.24 ± 5.2%) in our sample, which could be explained by a more one-sided nature of soccer (kicking with the preferred leg) compared to the sports included in our sample. In the second study, the authors calculated the k_RFD–SF_ of knee extensors in young athletes and reported that 15% of subjects could be classified as asymmetric (Smajla et al., 2020). In the current literature, there is no data about differences in (a)symmetries using the RFD-SF/RFR-SF protocol between different sports groups or genders. Moreover, we added additional outcome symmetry measures regarding TP_RFD_ and TP_RFR_. Our results revealed significantly lower values of symmetry in males compared to females for k_RFR–SF_ (88.4 ± 8.6 vs. 90.4 ± 8.0%) and TP_RFR_ (90.9 ± 6.8 vs. 92.5 ± 6.0%) (Figure 4), while symmetry values for k_RFD–SF_ and TP_RFR_ were similar (Table 4). Moreover, we found a significant effect of sport. Tennis players showed higher inter-limb symmetries in k_RFR–SF_ (91.1 ± 7.7%) compared to basketball players (88.4 ± 8.7, *p* < 0.05, δ_R_ = 0.37) and students (87.6 ± 8.7%), while the same was found for TP_RFR_ (tennis: 93.1 ± 6.0%, basketball: 90.9 ± 6.7%, and students: 90.5 ± 6.7%). It is well established that daily tasks and sports activities require fine motor control of muscle contraction and relaxation (Kato et al., 2019). This is the first study that evaluated symmetry outcome measures using the RFD-SF/RFR-SF protocol between genders and different sports groups. It seems that the outcome parameters of relaxation phase of the contraction are more sensitive to detect differences between our gender and sports groups. This can be supported with the study of Ohtaka and Fujiwara (2019), in which it was shown that the control error from the desired target force level was significantly greater for muscle relaxation than for contraction. Furthermore, it seems that muscle relaxation is more specific than contraction. For example, a deficit in muscle relaxation has been demonstrated in novice badminton players compared to skilled players and can be reduced with training (Sakurai and Ohtsuki, 2000). If one of the legs is used less frequently during specific sports movements, its relaxation ability may be reduced which can explain lower symmetry values. Tennis players perform a substantial amount of lateral movement on both sides (preferred and non-preferred), while basketball players use one leg more frequently because of specialized playing roles, which could be the possible reason for lower symmetry values. Lower symmetry values in students compared to tennis players are difficult to explain as students perform a variety of sports activities which may have different influences on lower-limb symmetry abilities.

There are some limitations regarding the age and gender in our sample. Even though the strength of our study is a large number of participants, our sample size was not gender-balanced. Moreover, the students’ group was slightly older compared to basketball and tennis players, while their sports activities vary between participants which may have a different influence on inter-limb symmetry. Nevertheless, this was the first study in the field that compared inter-limb symmetries using the RFD-SF/RFR-SF protocol to examine gender and sports differences. To obtain more valuable information, future studies should compare a wider range of sports and compare the sensitivity of established outcome measures for (a)symmetry calculation relative to RFD-SF/RFR-SF outcome measures.

Evaluation of submaximal force production and relaxation is important for all functional and sports movements. Our results suggest good to excellent intra-session reliability of the reduced RFD-SF/RFR-SF protocol which could be used for inter-limb symmetry quantification. The advantage of the RFD-SF/RFR-SF protocol is that force production and relaxation ability are tested across a wide span of submaximal intensities. Moreover, the relaxation phase of muscle contraction is often overlooked, although it was shown to be more sensitive for detecting various impairments. The sensitivity of RFD-SF/RFR-SF-derived measures should be further explored in future studies to confirm their use for inter-limb asymmetry investigation.

## Data Availability Statement

The raw data supporting the conclusions of this article will be made available by the authors, without undue reservation.

## Ethics Statement

The studies involving human participants were reviewed and approved by the Slovenian Medical Ethics Committee (approval no. 0120-99/2018/5). Written informed consent to participate in this study was provided by the participants’ legal guardian/next of kin.

## Author Contributions

NŠ conceptualized the idea and overviewed the measurement procedures and administration. DS and JŽ collected the data. JŽ analyzed the collected data. DS wrote the manuscript. NŠ and JŽ finalized the manuscript. All authors contributed to the article and approved the submitted version.

## Conflict of Interest

NŠ was employed by the company S2P, Science to Practice d.o.o. The remaining authors declare that the research was conducted in the absence of any commercial or financial relationships that could be construed as a potential conflict of interest.
